# Birth related parameters are important contributors in autism spectrum disorders

**DOI:** 10.1038/s41598-022-18628-4

**Published:** 2022-08-22

**Authors:** Nilanjana Banerjee, Pallabi Adak

**Affiliations:** Manovikas Biomedical Research and Diagnostic Centre, Manovikas Kendra Rehabilitation and Research Institute for the Handicapped, 482 Madudah, Plot I-24, Sector J, E.M. Bypass, Kolkata, West Bengal 700107 India

**Keywords:** Psychology, Risk factors

## Abstract

Autism spectrum disorders is a group of childhood onset neurodevelopmental disorders affecting millions of children across the globe. Characterised by age inappropriate lack of reciprocal social interaction, repetitive behaviours and deficits in communication skills, it has been found to have genetic, epigenetic and environmental contributions. In this work, we wanted to identify the effects of birth related parameters on the disease pathogenesis in an exposed population of West Bengal, India. We have considered age of both parents at birth, difference in parental age, familial history of mental illness, delay in developmental-milestones, birth-weight, birth-order, birth-term, mode of delivery and gestational complications as contributors. We found the parental age and their age difference to be the most important contributors towards ASD in this population. Birth order, sex of the probands, complications during gestation, birth weight, family history of mental illness and birth history also contributed to the condition, although to a lesser extent. Since such types of data are lacking in Indian population, this report adds useful information to the relevant field.

## Introduction

Autism spectrum disorders (ASD) are a group of childhood onset neurodevelopmental disorders with multifactorial aetiology. It is characterised by age-inappropriate reciprocal social interactions, repetitive, stereotypic behaviours and communication deficits. ASD includes Asperger’s syndrome, autistic disorders, childhood disintrigative disorders and pervasive developmental disorders not otherwise specified (PDD-NOS)^1^. Several studies have confirmed significant genetic contributions and heritable nature of the disorders^2,3^. Epigenetic modifications have also been found to have significant contributions in the pathophysiology of the same^4,5^. In addition, studies have shown that environmental factors and birth related variables play significant roles in the etiology of ASD^6–10^. Since ASD is multifactorial disorder, interplay between genetic, epigenetic and environmental factors is quite expected in the disease pathology.

In a meta-analysis, Gardener et al.^8^ examined over 50 prenatal factors that would be associated with autism risk including advanced paternal age, medications used during pregnancy, bleeding, gestational diabetes, previous foetal loss, hypertension and preeclampsia. In another study, in addition to the parental age, grandparental age (when they gave birth to the parents of the affected children), also turned out to be the risk factors for the same^9^. Too low or too high parental age were found to be risk factors for autism in other studies also^6,10^. Assisted reproductive technology (ART) which accounts for about 1–3% of birth in the present days, have been associated with low birth weight and congenital defects due to the stress on the central nervous system^11^. Iron deficiency of the pregnant mothers have also been associated with impairment in the development of cognitive, motor and language skills and might lead to ASD^12^. In-utero or early life exposure to environmental pollutants like heavy metals, phthalates, polychlorinated biphenyls (PCBs), air pollutants and pesticides have been also associated with ASD^13^. Certain medications of the pregnant mothers like antidepressants^14^ or anti-seizure medications ^15^ have been linked to ASD as well. Moreover low birth weight^16^, methods of delivery, familial history of mental disorders^17^ and maternal immune activation during pregnancy^18^ have lead to the birth of children who later developed ASD.

In the present work, we have studied the birth related variables in 170 cases of ASD in the children of West Bengal, India. Attempts have been made to identify the risk factors for ASD in these children in terms of age of the parents at birth, difference in parental age, familial history, delay in developmental milestones, birth weight, birth order, birth term, mode of delivery and gestational complications of the mother. Although birth-related variables have been reported to be risk factors for ASD in previous studies, similar findings are rare in the Indian population. So we made an attempt to identify such factors associated with ASD in this population from the demographic data collected during recruitment of the study participants.

## Results

### Demographic charactertics of the study participants

170 individuals with autism spectrum disorders (ASD) and their parents were recruited as the study participants. These children were chosen on the basis of DSM-IV criteria and had a mean CARS score of 34.68 ± 3.96. Average age of the probands was 6.13 ± 3.67 years with a male: female ratio of 6.39: 1. In this study, 76.47% were first-born children while 21.18% were second-born children and 2.35% were born as the third child. Average age of the mothers at the time of birth was 28.38 ± 4.41 years and that of the fathers was 34.87 ± 5.00 years, with an average parental age difference of 6.48 ± 3.64 years. Mean birth weight of the probands was 2.81 ± 0.58 kg. About 80% were full term babies, 19% were premature babies and 1% were hyper mature babies. Most of the children were born via Caeserean section (68.24%), while the rest were either born by spontaneous vertex delivery (25.3%) or via forceps delivery (6.47%). About 46.5% of the mothers had gestational complications like anaemia, urinary tract infections, injury due to falling down or domestic violence, fever, bleeding during pregnancy among many reasons (details of such complications are given in the Supplementary raw data), while the rest did not have any such problems. However, birth route did not contribute to birth complications (Fig. 2a viii). On analysing their family history it was found that about 59% of the families had history of mental illness while the rest did not. Previous studies have confirmed genetic contributions and heritable nature of such disorders^3,5^. So it might be quite expected that such problems (related mental illness/retardation) would exist in their families also. We found that, problems in the family members of the study participants were varied ranging from mental retardation, depression; delay in speech as found in autism, autism, epilepsy, schizophrenia, aggressive behaviours, reclusiveness, hyperactivity etc. (details are given in the Supplementary raw data). About 85% of the children had a delay in developmental milestones while the rest 15% did not have the delay in developmental milestone but they developed ASD at a certain point of time.

### Principal component ananlysis

**Figure 1:**
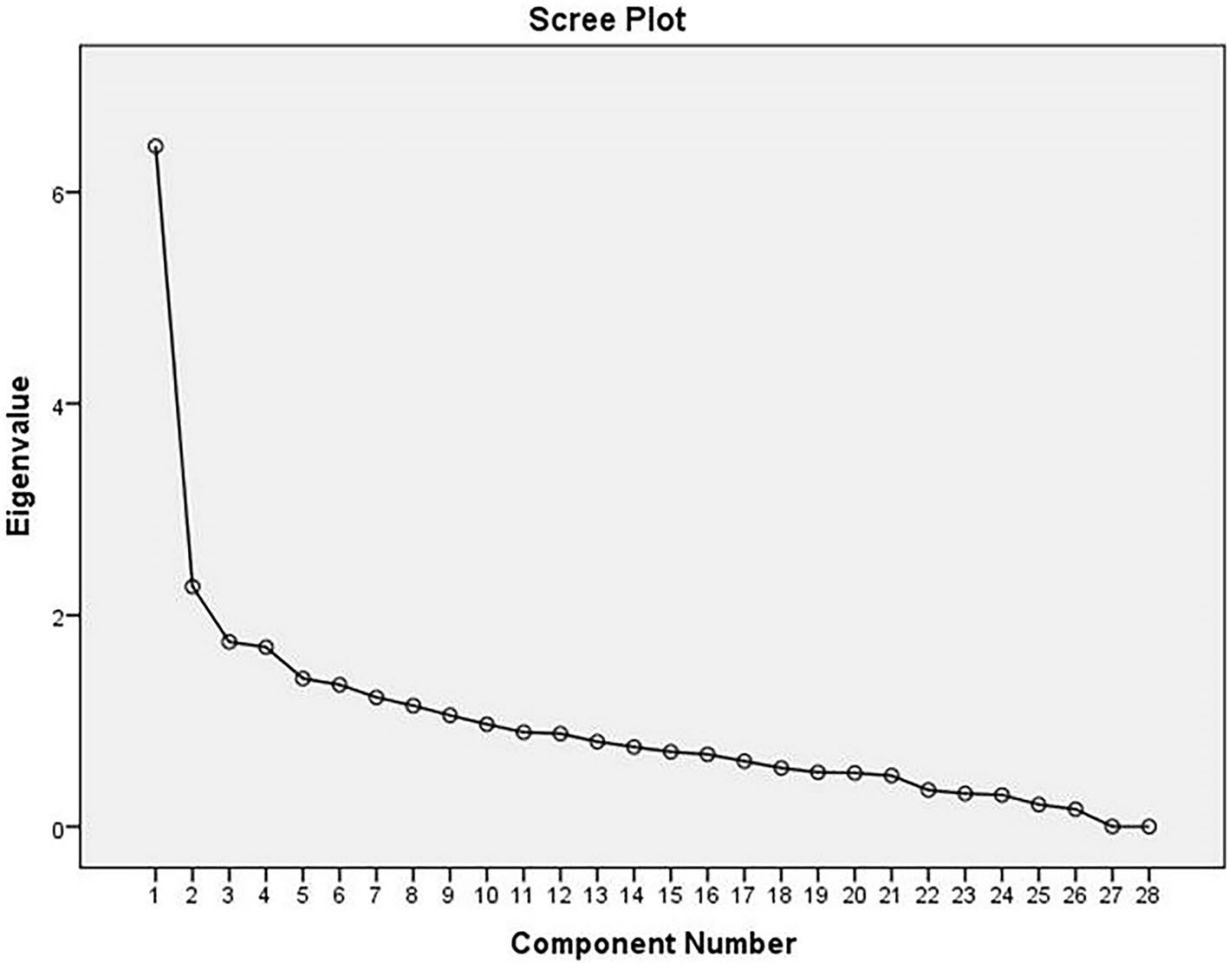
Scree plot of Eigen values for the data set.

Principal component analysis, or PCA, is a dimensionality-reduction method that is often used to reduce the dimensionality of large data sets that still contains most of the information in the large set^19^. In other words, the goal of running a PCA is to reduce the number of variables of a data set, while preserving as much information as possible. We did PCA for our dataset in the present work by employing SPSS software. Test for sphericity (Table S1) was significant for the data set (*p* = 0.009) but the sampling adequacy was low (0.502) so, Varimax Rotation model was used for the primary analysis. The extracted communalities by the corresponding models are shown in Table 1. More than 60% variance was shown for majority of the components including, age of both parents, difference in parental age, birth history, family history, developmental milestone, age and sex of the probands. The component matrix analysis extracted the traits into 9 components (Table 2). The first component had significant contributions from overall CARS score, general impression, relating to people, emotional and visual response, imitation, object use, verbal and nonverbal communications, listening response level and consistency of intellectual response. Significant contributions from father’s and mother’s age at the time of birth, difference in parental age and birth order were found under component 2. Mother’s age at the time of birth and taste, smell and touch response had significant contributions under component 3. Birth term, sex of the probands, complications and medications during pregnancy /child birth had important contributions under component 4. Again, birth weight was important contributors under component 6. Then the components were further subjected to Varimax rotation model with Kaiser Normalization which further displaced them as is shown in Table 3. Now we find that first component had significant contributions from CARS score, general impression, relating to people, emotional and visual response, imitation, object use, verbal and nonverbal communications, listening response level and consistency of intellectual response. Age of both parents and birth order are important contributors under component 2. Difference in parental age has a very significant level of contribution under component 4. Age, family history and birth history are important contributors under components 7, 8 and 9 respectively. Analysis of variance caused by each component showed greater than 50% contributions by the first six components (Table S2). The Scree plot (Fig. 1), shows that the component 1 has the highest Eigen values followed by components 2, 3 and 4 which is followed by the rest. Considering both the above together, we can say that variables present in the first 4 components are most significant for our data set. Although birth weight, family history of mental illness and birth history, belong to the later components but they also have higher Eigen values. Increasing of sample size might shift these parameters in the former components and confirm their significant contributions as found in studies of other populations.

**Table 1:** Communalities of the variables obtained from the Varimax rotation model.

	Initial	Extraction
**Communalities**		
CARS	1.000	0.989
Developmental milestone	1.000	0.668
Sex	1.000	0.629
Mother's age at time of birth	1.000	0.836
Father's age at time of birth	1.000	0.923
Difference in parental age	1.000	0.756
Complications and medications during pregnency/child birth	1.000	0.550
Birth weight (kg)	1.000	0.503
Birth order	1.000	0.537
Birth history	1.000	0.780
Birth term	1.000	0.599
Family history	1.000	0.635
Relating to people	1.000	0.680
Imitation	1.000	0.602
Emotional response	1.000	0.620
Body use	1.000	0.569
Object use	1.000	0.488
Adaption to change	1.000	0.617
Visual response	1.000	0.674
Listening response	1.000	0.740
Taste, smell and touch response and use	1.000	0.525
Fear or nervousness	1.000	0.496
Verbal communication	1.000	0.698
Nonverbal communication	1.000	0.626
Activity level	1.000	0.646
Level and consistency of intellectual response	1.000	0.517
General impression	1.000	0.724

Extraction method: principal component analysis.

**Table 2:** Component matrix^a^ table.

	Component								
	1	2	3	4	5	6	7	8	9
CARS	0.987								
General impression	0.797								
Nonverbal communication	0.775								
Listening response	0.774								
Verbal communication	0.770								
Relating to people	0.735								
Emotional response	0.661								0.324
Imitation	0.630								
Visual response	0.598				0.364	− 0.383			
Object use	0.582								
Level and consistency of intellectual response	0.510								
Father's age at time of birth		0.871							
Mother's age at time of birth		0.639	0.580						
Birth order		0.636							
Difference in parental age		0.423	− 0.591						
Taste, smell and touch response and use	0.395		0.478	0.315					
Body use			− 0.471				0.349		
Birth term				0.634		− 0.304			
Sex				0.502				0.436	
Complications and medications during pregnancy/child birth				0.502	0.396				
Fear or Nervousness		− 0.344		0.355					
Activity level					− 0.556				
Birth weight (kg)						0.590			
Adaption to change	0.381					0.382			
Developmental milestone					0.315		0.575		
Birth history					0.375			− 0.639	
Family history					0.301			0.308	0.462

Extraction method: principal component analysis.

^a^9 components extracted.

**Table 3:** Rotated component matrix^a^ table.

	Component								
	1	2	3	4	5	6	7	8	9
CARS	0.893		0.349						
Listening response	0.836								
Relating to people	0.778								
Nonverbal communication	0.771								
Verbal communication	0.737		0.301						
Visual response	0.729								
General impression	0.708			0.316					
Emotional response	0.699								
Imitation	0.620				0.377				
Object use	0.489		0.390						
Level and consistency of intellectual response	0.391								
Father's age at time of birth		0.889		0.327					
Mother's age at time of birth		0.863							
Birth order		0.551			− 0.331				
Adaption to change			0.744						
Taste, smell and touch response and use			0.416					0.307	
Fear or Nervousness			0.385			− 0.357			
Difference in parental age				0.804					
Body use				0.575					
Activity level					0.744				
Complications and medications during pregnancy/child birth					− 0.399			0.363	0.327
Birth term						− 0.672			
Birth weight (kg)						0.643			
Developmental milestone							− 0.793		
Family history								0.756	
Sex				0.362				0.539	
Birth History									0.872

Extraction method: principal component analysis. rotation method: Varimax with Kaiser Normalization.

^a^Rotation converged in 9 iterations.

### Correlation analysis

**Figure 2:**
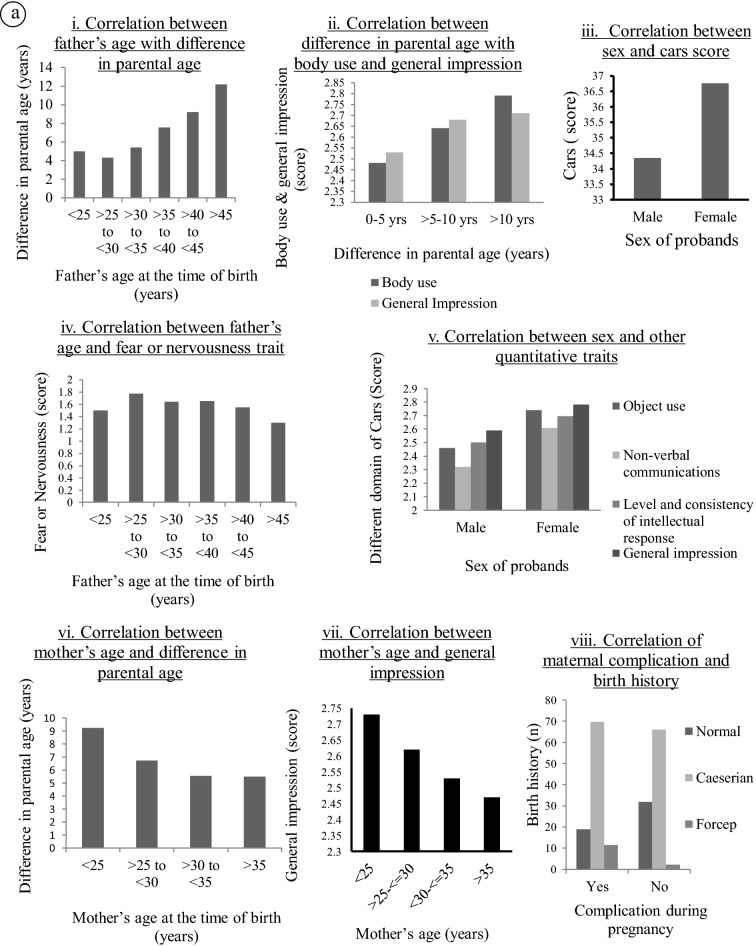

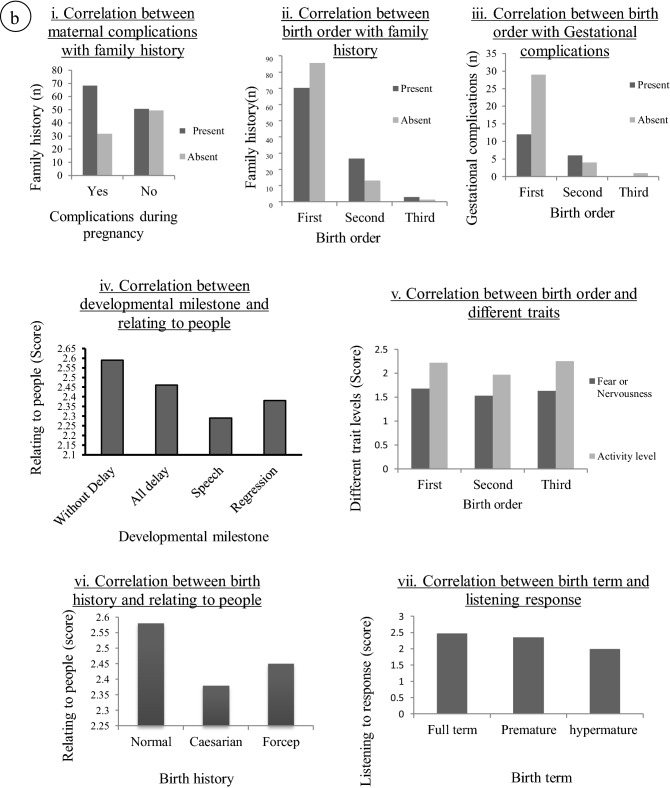
(**a**,**b**) Correlation between different parameters.

Pearson’s correlation test was employed to find correlation between continuous variables and Spearman’s Rank correlation test was done to find the correlation between ordinal variables. Results are shown in Table 4. We found significant positive correlation between father’s age and mother’s age at the time of birth and father’s age at time of birth-difference in parental age; difference in parental age-body use; difference in parental age-general impressions. Significant negative correlation was found between mother’s age at the time of birth-difference in parental age; mother’s age-general impressions; and father’s age-fear or nervousness. Spearman rank correlation test showed significant positive correlation between the sex of the probands and CARS score, general impression, object use, nonverbal communication and level of consistency. Significant positive correlation were also found between complications and medications during pregnancy/child birth with birth history and family history; between birth order and family history (*p* < 0.05). Significant negative correlations were found between developmental milestone–relating to people; birth history-relating to people; birth term-listening response, birth order-fear or nervousness and gestational complications and activity levels. Figure 2a(i–viii) and b(i–vii) gives a glimpse of such findings when the data has been stratified.

**Table 4:** Correlation table.

Variables	Correlation coefficient	*p* value	Type of test
Mother’s age–father’s age	0.708	< 0.01	Pearson's
Mother’s age–difference in parental age	− 0.240	0.002	
Mother’s age-general impression	− 0.184	0.016	
Father’s age–difference in parental age	0.516	< 0.01	
father’s age-fear/nervousness	− 0.176	0.022	
Difference in parental age-body use	0.188	0.014	
Difference in parental age-general impression	0.209	0.006	
Sex-nonverbal communications	0.184	0.016	Spearman’s
Sex-level and consistency	0.161	0.036	
Sex-general Impression	0.153	0.047	
Sex-CARS	0.172	0.025	
Sex-object use	0.184	0.016	
Developmental milestone-relating to people	− 0.182	0.017	
Birth history-relating to people	− 0.157	0.041	
Birth history-complications	0.199	0.009	
Birth term-listening response	− 0.154	0.045	
Birth order-fear and nervousness	− 0.158	0.040	
Birth order-family history	0.176	0.022	
Complications-activity level	− 0.157	0.041	
Complications-family history	0.194	0.011

### Regression analysis

Multiple regression analysis was done including all the predictors like father’s age, mother’s age, difference in parental age, birth order, birth history, birth weight, birth term, family history, complications and medications during pregnancy/child birth. Fifteen different models were analyzed based on 15 outcome variables. Significant results are shown in Table 5. Each model showed independent observations and absence of multi-co-linearity (having the following values: Dabrin Watson [DW] 1.5–2.5 and 1 < VIF < 10). Significant models were obtained for CARS score (*p* = 0.006), General Impression (*p* < 0.05), Relating to people (*p* = 0.32), Body use (*p* = 0.014) and Object use (*p* = 0.003). From the trait scores (T) scores we find that positive influence of sex was found on the overall CARS score. Lower mother’s age and higher difference in parental age were associated with higher scores for general impression. Here also positive influence of the sex of the probands was found on General impression. Relating to people was negatively influenced by the developmental milestone. Positive influence of difference in parental age was associated with higher trait scores for body use. Other models did not show any significant correlation.

**Table 5:** Regression analysis table.

Name of model	Variable	*p* value	F test score	DW test value	VIF	β (95% CI for β)	T score (p)
CARS	Sex	0.006		1.784	1.139	0.209 (0.69–4.125)	2.767 (0.006)
General impression	Mother’s age	0.016	5.87	1.580	1.292	− 0.184 (− 0.032 to − 0.003)	− 2.42 (0.016)
	Sex	0.042	4.20	1.523	1.139	0.156 (0.007 to 0.374)	2.050 (0.042)
	Difference in parental age	0.006	7.64	1.557	1.163	0.209 (0.007 to 0.041)	2.764 (0.006)
Relating to people	Developmental milestone	0.032	4.67	1.403	1.078	− 0.164 (− 0.148 to − 0.007)	− 2.16 (0.032)
Body use	Difference in parental age	0.014	6.18	1.698	1.163	0.188 (0.006 to − 0.050)	2.487
Object use	Sex	0.018	5.681	1.916	1.139	0.181 (0.047–0.506)	2.838 (0.018)

## Discussion

ASD is a neurodevelopmental disorder whose frequency is increasing at an alarming rate throughout the world including India. Genetic, epigenetic and environmental factors have found to play significant roles in the disease pathophysiology. In this work, we wanted to find out the role of birth related variables in the aetiology of ASD in our study population, the Indo-Caucasoid population of West Bengal, India. We have taken into account age of both parents at birth, difference in parental age, birth weight, birth term, birth order, mode of delivery, age, sex, familial history of mental illness, gestational complications/medications of the mother, and developmental milestones, for our study.

Principal component analyses of our data showed that age of both the parents’ at birth, difference in parental age, birth order, birth term, sex, complications during pregnancy were more important contributors to autism. Birth weight, developmental milestones and family history of mental illness also contributed to the disease phenotype although to a lesser extent (Table 2). More stringent analyses (Rotated Component matrix in Table 3) showed that age of both the parents at birth and difference in parental age still remained the most important contributors to the disease associated phenotype. We found (Table 4) that both the parents’ age was positively correlated with each other. Mother’s age was found to be negatively correlated with difference in parental age, indicating that younger the mother the more is the difference in parental age. We have also found for younger mothers there was negative impact on one of the trait scores ‘General impression’. Father’s age was positively correlated with difference in parental age, indicating older fathers contributed to the increased difference in parental age. Difference in parental age contributed significantly to two of the traits ‘body use’ and ‘general impression’ as expected, indicating greater is the difference in parental age greater is the severity in phenotypes exhibited. Age of the parents has been found to be important contributors to autism in other studies also. In a study by Wu et al.^20^, advanced parental age was found to be associated with an increased risk of autism in the offspring. In another study, it was found that older fathers contributed to highest severity of autism in the male child, while the mother’s age did not show any significant effect^21^. Elsewhere, it was found that both advanced parental age and increase in difference of parental age was related to increased risk of autism^22^. Another study found that in addition to many other factors lower maternal age contributed to significant risk of ASD^23^. All these are consistent to the findings in our study population. Older fathers are at risk of giving birth to a child with autism which may be due to the accumulation of spontaneous mutations in their sperm, which increases with age. Age of the mother has been found to be a confounding factor for autism also. Both younger mothers^22^ and older mothers^7^ are at higher risk of having a child with autism. Increased spontaneous mutation (although lesser rate than in sperms) might be the reason for older age mothers, but for younger age mothers the reasons are not very clear.

Birth order also contributed significantly, being in the second component. Birth order of the child has been previously associated with ASD phenotypes^24^. They found adaptive functioning and intelligence scores and mental retardation decreased with increasing birth order. We found that for increasing in birth order children had more severe phenotypes of autism (CARS ≥ 35), while about the first born children showed less severe phenotypes (CARS < 35). Birth weight, developmental milestone, family history and birth history also contributed but to a lesser extent.

As already known that autism has a sex bias and males are almost 5 times more susceptible than the females^25^. For us the ratio was 6.39:1 (male:female). We found sex of the probands were positively correlated with the overall CARS score, non-verbal communication, level and consistency, object use and general impression. Interestingly, on separating the males from the females it was found that the males have a lower mean CARS score (34.35) as compared to the females (36.76), indicating disease severity was more in the females than in the males, even though the frequency of occurance is much less in the females. This is similar to a previous observation found in another population of the same state^26^. They showed that, higher platelet/plasma 5-HT and plasma 5-HIAA in the females could probably increase the threshold level of ASD phenotypes in the females, thereby contributing to more severe phenotypes.

In our study, developmental milestone was negatively correlated with ‘relating to people’, indicating that developmental delay is associated with autism as found in previous studies^27,28^. Developmental delay may consist of global delay in reaching all the milestones or speech delay or regression where the children begin to develop normally until a point when regression of speech/other behavioral patterns occur. In our study population we came across all the three types of delay: about 46.47% had over all developmental delay, 18.83% had speech delay and 19.41% had regression. Similarly birth history, birth term, birth order and complications during gestation period had influence on phenotypic traits like relating to people, listening response, fear or nervousness, and activity level. Here, ‘Complications’ consisted of any type of illness of the mother during gestation/child birth including mostly anemia, fever, depression, bleeding, falling from certain height or other accidents, increase or decrease of blood pressure, jaundice, thyroid problem, hypoxia during child birth and medications for any of the above reasons. Some of the mothers had maintained very poor diet because they could not eat/vomited frequently during pregnancy. Our results are in agreement with a meta analysis which showed that the prenatal factors associated with autism risk were gestational hypertension, gestational diabetes, threatened abortion, and antepartum hemorrhage in addition to the parents’ age^29^. Maternal immune activation and dietary intake were also previously associated with ASD^30^. In our study, complications in in utero conditions and birth histories (spontaneous vertex delivery, forceps delivery or caesarian sections) were found to be important contributors as expected. Such complications were also found to be positively correlated with family history of mental illness. Complications were found to have a negative impact on the trait ‘activity level’.

From regression analyses (Table 5) it was found that there was linear relation between six of the traits of the CARS score (among 15) and age of the mother, difference in parental age, developmental mile stone and age and sex of probands. For other traits, linear relationships were not found although they were related as discussed above.

## Conclusion

In conclusion, we can say that our entire data analyses indicated that age of the parents and difference in parental age contribute primarily and most importantly to the disease phenotype in ASD. Birth order, sex of the probands, complications in mother’s health during gestation period and developmental milestones also contribute significantly to the disease severity. Birth weight, family history of mental illness and birth history also contributed to the condition, but to a lesser extent. Further studies involving larger sample size might throw some light on these aspects as well. To our knowledge, this is the first report of its kind in the ASD population of West Bengal, India.

## Methods

### Selection of study participants and collection of demographic details

A total of 170 children with ASD (147 males and 23 females), aged 3–15 years were included in the study. They were recruited from the out-patient department (OPD) of Manovikas Kendra, Kolkata based on DSM-IV criteria^31^ by an expert psychiatrist and clinical psychologists; symptom severity being assessed using the Childhood Autism Rating Scale (CARS). Informed written consent was obtained from the parent or caregivers. Children with gross chromosomal abnormalities and/or any other developmental, neurological or psychiatric conditions were excluded from the study. Details of birth related variables, including birth weight, birth term, birth order, mode of delivery, age, sex, familial history of mental illness, age of both parents at birth, gestational medications/complications and developmental milestones were noted based on structured questionnaires. The study was approved by the Institutional Human Ethical Committee of Manovikas Kendra. All methods were performed in accordance with the relevant guidelines and regulations.

### Scoring method

For statistical calculations, male individuals were assigned a score of 1 and female individuals were assigned a score of 2. Order of birth was scored as 1, 2, and 3 for first, second and third child respectively. Score of 1 was assigned for full term delivery and score of 2 and 3 were given to pre-term and post-term deliveries. Spontaneous vertex delivery was scored as 1, caesarean sections were scored as 2 and forcep delivery was scored as 3. Cases with no complications during pregnancy/at birth was scored as 0 and scored as 1 for showing any complication during pregnancy/at birth. Probands without family history of mental illness were given a score of 0 and with family history were given a score of 1. While considering developmental milestones, probands with no developmental delay were in group 0, while those with overall developmental delay were under group 1, those with speech delay were under group 2, and those with regression were grouped under group 3. Age of both parents at birth were sub divided under the following groups: Mother’s age at the time of birth were stratified into 4 groups: group 1: ≤ 25 years, group 2: > 25 to ≤ 30 years, group 3: > 30 to ≤ 35 years, and group 4: > 35 years. Father’s age at the time of birth were stratified in to 6 groups: ≤ 25 years; > 25 to ≤ 30 years, > 30 to ≤ 35 years, > 35 to ≤ 40 years. > 40 to ≤ 45 years and > 45 years. Birth weight of the probands were divided in to 4 groups: ≤ 1.5 kg under group1; > 1.5 kg to 2.5 kg, under group 2; > 2.5 kg to 3.5 kg under group 3; > 3.5 to 4.5 kg under group 4.

### Statistical analyses

Statistical analyses was done using the SPSS Software version 20, checking necessary assumptions before running the appropriate program. Principal component analysis was performed extracting important variables (in from of components) from a large set of variables which were available in dataset. Before running this dataset initial check was done for sphericity and adequacy of sampling for the dataset (Table S1).

## Supplementary Information

Supplementary Information.

## Data Availability

All data generated or analysed during this study are included in this published article and its supplementary information files.
