# Supplementary material for: Birth related parameters are important contributors in autism spectrum disorders
Source: Sci Rep. 2022 Aug 22;12:14277. doi: 10.1038/s41598-022-18628-4 (PMC9395415; doi:10.1038/s41598-022-18628-4)
Supplement: Supplementary file 1 — Supplementary Information. [file 41598_2022_18628_MOESM1_ESM.pdf]

Supplementary Information

Tile: Birth related parameters are important contributors in Autism Spectrum Disorders

Authors: Nilanjana Banerjee* & Pallabi Adak

Institutional Affiliation:

[1] Manovikas Biomedical Research and Diagnostic Centre, Manovikas Kendra Rehabilitation and Research Institute for the Handicapped, Kolkata-700107, West Bengal, India

Email of Corresponding author: 2006.nilanjana@gmail.com

Table S1 KMO and Bartlett's Test

Kaiser-Meyer-Olkin Measure of Sampling Adequacy.		.502
Bartlett's Test of Sphericity	Approx. Chi-Square	48.897
	df	28
	Sig.	.009

Table S2 Total Variance Explained

Component	Initial Eigenvalues			Extraction Sums of Squared Loadings			Rotation Sums of Squared Loadings		
	Total	% of Variance	Cumulative %	Total	% of Variance	Cumulative %	Total	% of Variance	Cumulative %
1	6.435	22.981	22.981	6.435	22.981	22.981	5.835	20.841	20.841
2	2.270	8.108	31.089	2.270	8.108	31.089	2.137	7.631	28.472
3	1.747	6.241	37.330	1.747	6.241	37.330	1.760	6.285	34.757
4	1.697	6.062	43.392	1.697	6.062	43.392	1.702	6.077	40.834
5	1.401	5.003	48.395	1.401	5.003	48.395	1.539	5.496	46.330
6	1.341	4.790	53.185	1.341	4.790	53.185	1.473	5.262	51.593
7	1.222	4.365	57.550	1.222	4.365	57.550	1.321	4.718	56.311
8	1.144	4.084	61.634	1.144	4.084	61.634	1.299	4.640	60.951
9	1.053	3.760	65.394	1.053	3.760	65.394	1.244	4.443	65.394
10	.968	3.457	68.851						
11	.893	3.188	72.038						
12	.879	3.141	75.179						
13	.803	2.868	78.047						
14	.753	2.690	80.737						
15	.708	2.527	83.264						
16	.684	2.441	85.705						
17	.619	2.210	87.914						
18	.553	1.976	89.891						
19	.513	1.834	91.725						
20	.507	1.811	93.535						

21	.481	1.720	95.255							
22	.345	1.232	96.487							
23	.312	1.115	97.602							
24	.299	1.067	98.669							
25	.208	.744	99.413							
26	.164	.585	99.998							
27	.001	.002	100.000							
28	6.187E-016	2.210E-015	100.000							

Extraction Method: Principal Component Analysis.

Raw data used for analyses

Sample no.	CARS Score	Developmental milestone [a]	Age (years)	Sex [b]	Mother's age at time of birth (years)	Father's age at time of birth (years)	Difference in parental age (years)	Complications and medications during pregnancy/ child birth [c]	Birth weight (kg)	Birth order [d]	Birth History [e]	Birth term [f]	FAMILY HISTORY [g]
1	38	0	9	1	30	40	10	0	1	2	2	1	1
2	36	1	5	1	30	40	10	0	0	1	2	1	1
3	35	1	9	1	36	41	5	0	2	2	2	1	1
4	39.5	1	9	1	26	31	5	0	2.2	3	1	1	0
5	35.5	1	2.5	1	30.5	31.5	1	0	0.75	1	1	1	1
6	40	1	5	1	20	25	5	0	3	1	3	1	1
7	39.5	0	4	1	36	43	7	0	3	2	1	1	0
8	37.5	3	6	1	28	30	2	0	3	1	1	1	1
9	34.5	0	4	1	24	28	4	1	2.5	1	2	2	0
10	33	1	4	1	22	28	6	1	3.2	1	2	1	0
11	38.5	1	5.5	1	26.5	36.5	10	0	3.5	1	1	1	0
12	39	1	7	1	28	35	7	1	3	1	1	2	1
13	38	3	7	1	27	32	5	1	2.8	2	1	1	1
14	39.5	0	11	1	28	37	9	1	2.25	2	1	1	0
15	38	3	10	1	30	37	7	1	2.5	1	2	1	0
16	38	0	6	2	23	35	12	1	2.5	2	1	1	1
17	31	1	5	1	26	28	2	0	2.5	1	2	1	1

18	31	1	9	1	31	36	5	0	2.1	2	1	2	1
19	37	2	6.5	1	31.5	43.5	12	1	2.5	1	2	1	0
20	30.5	0	7	2	29	35	6	1	4.2	1	2	1	1
21	30	1	7	1	28	37	9	0	1.4	1	1	1	0
22	34.5	1	12	1	34	39	5	0	3.5	2	1	1	1
23	37	0	2.5	1	27.5	30.5	3	0	2	1	2	2	1
24	34.5	1	6.5	2	30.5	35.5	5	0	2.2	1	1	1	0
25	37.5	1	3	1	37	47	10	1	3.5	1	2	1	0
26	36.5	0	7	1	25	33	8	0	2.6	1	1	1	0
27	38	1	2	2	26	41	15	1	3.325	1	3	2	1
28	30	2	4	1	29	32	3	0	3.1	1	2	1	1
29	42.5	1	8	2	27	39	12	1	2.7	1	1	1	1
30	42	1	3	1	30	44	14	0	2.5	1	2	1	1
31	30	1	3	2	33	39	6	1	3.69	1	2	2	0
32	43	1	4	1	30	34	4	1	2.8	1	2	1	1
33	32.5	0	25	1	23	28	5	1	3	1	3	1	1
34	42	1	8	1	25	31	6	0	2.4	1	2	1	0
35	30	1	7	1	28	36	8	1	2.5	2	2	3	1
36	40.5	3	2.1	1	31.9	33.9	2	1	3.25	1	2	1	0
37	40.5	2	4	1	26	33	7	0	3.3	1	1	1	0
38	30	2	7	1	23	31	8	0	3.1	1	1	1	1
39	40	2	4	1	27	37	10	1	3.6	1	2	2	0
40	33	2	9	1	29	39	10	0	3	2	2	2	0
41	36	1	5	1	29	39	10	0	2.5	1	2	2	1
42	30.5	3	3	1	29	37	8	0	2.5	1	2	1	0
43	34.5	2	5	1	27	30	3	0	2.7	1	1	1	0
44	32	1	10.5	2	32.5	32.5	0	1	2.25	1	2	1	1
45	34	2	3	1	31	37	6	0	2.7	1	2	1	0
46	30.5	1	6	1	21	29	8	0	2.6	1	2	1	0

47	34.5	1	5	1	30	36	6	1	2.5	1	2	1	0
48	30	1	6.6	1	24.4	33.4	9	0	2.9	1	2	1	1
49	34.5	0	5	1	25	30	5	0	2.6	1	1	1	0
50	35	2	6	1	24	30	6	0	2.5	1	1	1	0
51	40.5	2	10	1	30	35	5	1	2.2	1	1	1	0
52	34	0	7.5	1	29.5	29.5	0	0	3.5	1	2	1	0
53	33	3	4	1	25	35	10	0	3.5	1	2	1	0
54	30	1	5.6	1	36.4	39.4	3	1	3.5	3	2	2	1
55	33	1	3	1	23	30	7	0	2.5	1	2	1	1
56	40	1	11	1	25	35	10	0	2	1	3	2	0
57	35	0	5	1	31	36	5	0	2.8	1	1	1	1
58	30	2	6.7	1	29.3	38.3	9	1	3	1	2	1	1
59	31.5	2	6	1	27	35	8	0	3.63	1	1	1	1
60	42	2	7	1	27	30	3	0	2.75	1	1	1	1
61	30	3	8	1	22	28	6	1	2.85	1	3	1	1
62	34	2	7	1	32	33	1	0	3	2	2	2	1
63	38	1	6	1	33	34	1	0	2.7	1	1	1	0
64	36.5	2	5.16	1	30.84	34.84	4	0	4	2	2	1	1
65	32.5	0	4.83	1	22.17	30.17	8	1	2.7	1	3	1	0
66	30	1	8	2	32	48	16	1	2.5	2	2	2	1
67	36.5	1	5	1	25	33	8	1	3.6	1	1	1	0
68	36	0	3.9	1	28.1	36.1	8	0	2.5	1	1	1	0
69	36	1	5	1	27	39	12	0	2.75	2	2	2	0
70	31	1	3.7	1	36.3	47.3	11	0	3	1	2	1	1
71	30	0	6	2	28	29	1	0	2.65	1	1	1	1
72	30.5	1	2.1	1	26.9	36.9	10	1	2.3	1	2	1	0
73	30	0	7	1	26	41	15	0	2.9	1	2	1	0
74	35.5	1	5	1	24	29	5	0	2.85	1	2	1	0
75	35.5	1	5	1	26	37	11	0	2.1	2	1	1	0

76	30	0	8	1	26	32	6	1	2.5	1	2	2	0
77	38.5	1	6	2	19	36	17	1	2.5	1	1	1	1
78	31	1	19.6	1	33.4	44.4	11	1	3.1	1	2	1	1
79	37.5	3	3	1	28	29	1	0	2.4	1	2	1	1
80	35	0	5	2	22	30	8	1	2.3	2	1	1	1
81	36	1	5.5	1	29.5	32.5	3	1	2.5	1	2	1	1
82	36.5	0	11	1	27	31	4	1	2.75	2	2	1	1
83	37	1	3	1	24	34	10	1	2.75	1	2	1	0
84	34.5	2	8	1	27	35	8	1	2.25	1	3	2	1
85	38.5	1	5	1	30	37	7	1	2.9	2	3	1	1
86	35.5	3	2.9	1	21.1	29.1	8	0	2.7	1	2	1	0
87	30.5	2	4.5	1	30.5	35.5	5	1	3.5	1	1	1	1
88	34	1	3.9	1	26.1	31.1	5	0	2.7	1	2	1	1
89	36	2	6.8	2	20.2	26.2	6	1	2.9	1	2	3	0
90	31	3	9	1	30	33	3	1	2.35	2	1	1	1
91	30	3	7	1	34	46	12	1	2.5	2	2	1	1
92	30.5	2	4	1	30	32	2	0	2.7	1	2	1	0
93	30.5	3	7.4	1	37.6	49.6	12	1	3.4	3	2	1	1
94	32	2	3	2	22	27	5	0	3.1	1	2	2	1
95	31	1	7	1	36	44	8	1	2.8	2	2	1	1
96	30.5	3	3.5	1	38.5	44.5	6	1	3.5	2	2	2	1
97	30	3	12	1	31	35	4	1	2.55	2	2	1	1
98	34.5	1	4	1	23	27	4	1	3.2	1	2	1	1
99	50	1	7.7	2	30.3	34.3	4	0	2.5	1	2	2	1
100	33.5	1	10	1	26	37	11	0	3.8	1	2	1	1
101	31.5	1	4.11	1	31.89	40.89	9	1	3	1	2	1	0
102	31	1	7	1	28	35	7	1	2.5	1	2	2	1
103	32	1	3.7	1	31.3	40.3	9	1	2.5	1	2	1	1

104	38.5	0	3.1	1	29.9	37.9	8	1	3.5	1	2	2	1
105	32	1	2.11	1	34.89	34.89	0	1	2.9	2	2	1	1
106	33.5	1	3.5	1	26.5	32.5	6	1	4.08	1	2	1	1
107	36	1	2.6	1	27.4	40.4	13	0	2.7	1	2	1	1
108	41.5	0	3.4	1	42.6	44.6	2	0	2.56	2	2	1	1
109	30	0	7	1	27	28	1	1	3.1	1	2	1	1
110	38	3	2.3	2	27.7	32.7	5	0	2.4	1	2	1	1
111	34.5	0	13.3	1	29.7	37.7	8	0	2.9	1	1	1	1
112	35.5	2	15.6	2	20.4	33.4	13	1	3.5	2	1	1	1
113	38.5	1	6	1	31	42	11	1	2.75	3	2	1	1
114	39.5	1	3	1	32	38	6	1	2.65	1	2	1	1
115	30	1	13	1	37	42	5	0	3.5	2	2	1	1
116	31	3	3.5	1	31.5	31.5	0	1	2.75	1	1	2	1
117	43.5	2	2.11	1	28.89	29.89	1	1	2.8	1	3	1	1
118	40.5	0	4	2	27	31	4	0	3.2	1	2	1	0
119	37.5	1	4.1	1	33.9	36.9	3	0	2.2	1	2	1	0
120	31.5	1	10	1	28	34	6	0	2.8	1	2	1	0
121	40	0	3.3	1	37.7	40.7	3	0	2.6	1	2	1	0
122	42	3	3	2	20	29	9	0	3.5	1	2	1	1
123	40	1	5.1	2	26.9	30.9	4	1	2.45	1	2	1	1
124	30	2	4.3	1	34.7	37.7	3	1	2.5	2	2	1	1
125	35.5	1	4	1	26	32	6	0	2.6	1	2	1	0
126	36	3	2.5	1	22.5	27.5	5	0	2.05	1	2	1	0
127	38.5	1	6.5	1	22.5	32.5	10	0	2.82	1	2	1	0
128	38	1	4.5	1	31.5	40.5	9	0	3.4	1	2	1	1
129	36.5	1	7	1	23	34	11	1	3.2	1	2	1	1
130	38.5	1	5	1	19	24	5	0	2.8	1	1	1	1
131	38.5	1	4	1	31	39	8	0	3.3	1	2	1	0
132	30.5	2	4.2	1	29.8	41.8	12	0	2.8	1	2	1	1

133	33.5	2	3.4	1	36.6	37.6	1	0	2.2	1	2	1	1
134	38	1	5	2	29	41	12	0	2.8	2	1	2	0
135	30	1	15	1	30	35	5	0	3.8	1	1	1	0
136	32	1	3	1	25	32	7	0	3	1	2	1	0
137	29	1	13	1	38	43	5	0	3.4	2	2	1	1
138	36	3	2	1	26	30	4	0	3.5	1	2	1	0
139	33.5	1	12	1	24	37	13	1	3	2	1	1	1
140	30	1	6	1	23	30	7	1	3.25	1	3	1	1
141	31.5	3	5	1	28	33	5	0	3.2	2	2	1	1
142	32.5	3	2.3	1	35.7	38.7	3	0	3.5	1	2	1	1
143	36	1	1.7	1	25.3	31.3	6	1	2.5	2	2	1	0
144	38.5	3	5	1	27	31	4	0	2.9	1	2	1	1
145	40	3	10	2	33	38	5	1	4.8	2	2	1	0
146	41	3	2.3	1	31.7	31.7	0	1	2.8	1	2	2	0
147	39	3	3	1	22	28	6	0	3.5	1	2	1	0
148	31.5	2	5	1	27	28	1	1	2.9	1	2	2	0
149	40	3	2.4	2	27.6	32.6	5	1	2.9	1	2	1	1
150	30	1	6.6	1	29.4	36.4	7	1	2.7	1	2	2	1
151	29.5	1	20	1	24	28	4	1	1.9	1	3	1	0
152	30	2	2.1	1	32.9	36.9	4	1	3.3	1	2	1	1
153	31.5	3	2.6	1	27.4	30.4	3	0	2.75	1	2	2	0
154	31	1	3.7	1	33.3	37.3	4	1	2.5	1	2	1	1
155	34	3	8	1	26	32	6	0	2.75	1	2	2	1
156	34.5	2	4.8	2	22.2	35.2	13	0	2.4	1	1	2	1
157	32.5	2	2.5	1	24.5	34.5	10	1	2.4	1	2	2	1
158	32.5	3	5.1	1	27.9	37.9	10	1	2.35	2	2	1	1
159	33.5	1	13	1	29	37	8	0	2.8	1	2	1	1
160	30.5	3	2	1	30	31	1	1	2.9	1	2	1	1
161	30	3	5.6	1	30.4	35.4	5	0	3.7	1	1	1	0

162	33	3	3.1	1	26.9	28.9	2	0	2.7	1	2	1	0
163	30	1	4.4	1	30.6	40.6	10	1	3.5	1	2	1	1
164	29	3	14.6	1	25.4	30.4	5	1	3	1	2	1	0
165	31	2	5	1	30	35	5	0	2.5	1	2	1	0
166	30	2	14	1	24	26	2	1	3.5	1	2	1	1
167	33.5	2	7	1	22	33	11	0	2.6	1	2	2	0
168	30	1	4	1	34	35	1	0	2.3	1	1	1	1
169	35	1	9	1	34	37	3	1	2.6	1	1	2	1
170	33	0	12.5	1	30.5	40.5	10	0	2.5	2	2	1	0

[a] Developmental milestone = probands with no developmental delay were in group 0, while those with overall developmental delay were under group 1, those with speech delay were under group 2, and those with regression were grouped under group 3

[b] Sex = 1 for males, 2 for females

[c] Complication in utero/child birth = Cases with no complications during pregnancy/child birth was scored as 0 and scored as 1 for showing any complication during pregnancy/child birth

[d] Birth order = 1, 2, and 3 for first, second and third child respectively

[e] Birth History = Spontaneous vertex delivery was scored as 1, caesarean sections were scored as 2 and forcep delivery was scored as 3

[f] Birth term = Score of 1 was assigned for full term delivery and score of 2 and 3 were given to pre-term and post-term deliveries

[g] Family History = Probands without family history of mental illness were given a score of 0 and with family history were given a score of 1

Raw data continued….. details of complications of the mother and family history.

Sample no.	Complications and medications during pregnency/child birth	Complications and medications during pregnency/child birth (codes)	FAMILY HISTORY	FAMILY HISTORY (codes)
1	NONE	0	Developmental delay in uncle, Stereotypic behavior of mother	1
2	NONE	0	mother depressed since first child aborted	1
3	NONE	0	Brother of proband is suffering from Asperger's syndrome; Mental retardation in grandparents	1
4	NONE	0	None	0
5	NONE	0	Cousins are Mentally retarded	1
6	NONE	0	Grand mother's cousin has mental retardation	1
7	NONE	0	None	0
8	NONE	0	relatives of maternal side have mental retardation	1

9	child had bigger head circumference, mother slipped at age of nine months during pregnancy	1	None	0
10	iron supplimentation of anemia to mother,	1	None	0
11	NONE	0	None	0
12	she is subjected to domestic violence of father	1	Father depressive	1
13	birth asphyxia, breathing problem	1	father has mental reatrdation	1
14	at 6 months epileptic sizers started on anti epileptic drugs till 9 months	1	None	0
15	birth injury, prolonged labour pain	1	None	0
16	bleeding during 2nd month just preceding pregnancy	1	Cousin had delayed speech problem in childhood	1
17	NONE	0	Mother hyperactive; paternal grandfather reclusive	1
18	NONE	0	Maternal uncle dumb	1
19	mother fell down during pregnancy	1	None	0

20	gall blader stone detected during pregnancy	1	Maternal grandmother suffered from depression	1
21	NONE	0	None	0
22	NONE	0	Paternal grandfather had diabetes and neurological problems	1
23	NONE	0	Mother shows borderline mongoloid features; father obese	1
24	NONE	0	None	0
25	acidity	1	None	0
26	NONE	0	None	0
27	1 month bleeding of mother	1	Mental retardation and epilepsy in maternal uncle's daughter; Father with aggressive temperament	1
28	NONE	0	delayed speech problem in maternal aunt's daughter	1
29	skin disease, candida infection on the skin, birth asphyxia, had vomiting till 9 months,low bp	1	Mental reatrdation in maternal uncle's son and maternal granduncle's daughter	1
30	NONE	0	Paternal grandaunt was lunatic, grandmother hysteric, maternal, uncle had chronic paranoid schizophrenia	1

31	fever during 6 months, infection after delivery juvenile jaundice	1	None	0
32	Breathing trouble of mother just after birth	1	Delayed speech in elder sister	1
33	fell from a height during the initial period	1	Mother is mentally retarded; father's younger brother is suffering from cerebral palsy	1
34	NONE	0	None	0
35	fallen from the height several times and mild seizure occur	1	Paternal aunt is suffering from mental depression	1
36	Urinary tract infection, high fever 5 days after delivery	1	None	0
37	NONE	0	None	0
38	NONE	0	Cerebral palsy in paternal uncle's son	1
39	delayed birth cry	1	None	0
40	NONE	0	None	0
41	NONE	0	Delayed speech in elder sibling, father, paternal aunt; Father is epileptic	1
42	NONE	0	None	0
43	NONE	0	None	0
44	had accident during pregnancy (leg injury)	1	Maternal grand mother had schizophrenia;diabetes heart disease in family	1
45	NONE	0	None	0

46	NONE	0	None	0
47	epilepsy	1	None	0
48	NONE	0	Paternal grandfather's son suffered from mental disorder	1
49	NONE	0	None	0
50	NONE	0	None	0
51	prolonged cough & cold problem	1	None	0
52	NONE	0	None	0
53	NONE	0	None	0
54	CONVULSION OF THE MOTHER AT THE TIME OF BIRTH, HYPERTENSION OF MOTHER, MALNUTRITION, NEONATAL ASPHYXIA, NEONATAL JAUNDICE	1	Convulsion of mother at time of birth	1
55	None	0	Mental illness in paternal uncle	1
56	None	0	None	0
57	None	0	Maternal uncle has cerebral palsy	1

58	High blood pressure of mother during pregnancy, under pressure medication, abdominal pain in 6 months	1	Maternal uncle had delayed speech development; cousin is suffering from psychological problem; Paternal grandfather mentally retarded	1
59	None	0	Epilepsy in paternal aunt	1
60	None	0	Epileptic seizure in father's cousin	1
61	mother was extremely stressed during pregnancy	1	Father and paternal greatgrandmother had learning problems	1
62	None	0	Younger brother is suffering from autism	1
63	None	0	None	0
64	None	0	Delayed speech in elder brother, Father has eye contact problem	1
65	baby did not cry immediately after birth, convulsion once	1	None	0
66	diabetes	1	mother and grandmother suffering from epilepsy	1
67	High bp	1	None	0
68	None	0	None	0
69	None	0	None	0
70	None	0	Father is suffering from behavioural problem	1

71	None	0	Mother is suffering from severe mood swings and allergy	1
72	mother had hypoxia during birth of the chilld	1	None	0
73	None	0	None	0
74	None	0	None	0
75	None	0	None	0
76	mother had severe back pain and high bp	1	None	0
77	low blood pressure at 8 month pregnancy	1	Mothers cousin mentally retarded and on father's side mental retardation and physical deformity present	1
78	hypertension	1	Mother has problem in social mixing	1
79	None	0	Autism in maternal uncle; mental illness in paternal aunts brother	1
80	thyroid was detected at the age of 1.5 yr, measles at childhood, NO BIRTH CRY	1	epilepsy in mother's maternal cousin	1
81	mother had jaundice during pregnancy	1	Autim in maternal grandmother's sister	1

82	bleeding at 1.5 months of pregnancy, injection & medicine given	1	Maternal cousin had regressive speech at 3years age, aggressive behaviour and seizure; Most people on the paternal side are reclusive and of limited speech	1
83	sugar level was high	1	None	0
84	gastric problem, neonatal asphyxia	1	Epilepsy in mother's maternal cousin	1
85	did not cry after birth, hospitalised at 2.5 years for food poisoning, neonatal asphyxia	1	Mental retardation in maternal aunt's daughter and paternal aunt's daughter	1
86	None	0	None	0
87	medicine taken to sustain pregnancy since she had fibroid problem, has earlier miscarriage	1	Paternal cousin has autism, Cerebral Palsy; Maternal uncle has personality disorder	1
88	None	0	Mental retardation in paternal aunt's daughter; Cerebral Palsy in paternal uncle's grandson	1
89	jaundice at 3rd trimester	1	None	0
90	bleeding at 2 months of pregnancy thereafter spotting till 6 months	1	Paternal uncle has mental retardation	1

91	mother had thyroid problem, bleeding during first 2 months	1	Mother has hypothyroidism and mental retardation	1
92	None	0	None	0
93	hyperthyroidism during pregnancy	1	Paternal aunt mentally retarded	1
94	None	0	Delayed speech in father's cousin brother	1
95	mother had medication for controlling pressure	1	Paternal grandmother had seizure problem and maternal uncle had delayed speech	1
96	mother had two miscarriages, 2nd child died in the womb due to lack of fluid at very late stage	1	Paternal cousin has language disorder and paternal uncle has Schizophrenia	1
97	mother was under a lot of mental stress during pregnancy	1	Paternal uncle had mental retardation and convulsion; mental retardation present in father's maternal aunt	1
98	bleeding during first 2 months, mother had severe high bp	1	Mental illness in maternal grandfather and aunt; mental retardation in paternal grand uncles	1
99	None	0	Maternal uncle has Downs syndrome	1
100	None	0	Maternal aunt has poor IQ	1

101	mother had overian cyst and thyroid problem, 1st issue was misscarraged at 3rd month	1	None	0
102	massive diarrhea at early stage of pregnancy	1	Mental illness in paternal grand uncle	1
103	less movment of baby during pregnancy	1	Mental illness in paternal grandfather and uncle	1
104	1st miscarriage at 3 months	1	Maternal uncle had Down's syndrome	1
105	hypothyroid, jaundice	1	Father's maternal grandmother had depression	1
106	mother had vomiting ,unable to eat, all throughout pregnancy	1	Mother had depression throughout pregnancy	1
107	None	0	Mother's maternal aunt's son has Mental Retardation; Paternal uncle had delayed speech problem	1
108	None	0	Paternal uncle is suffering from mild schizophrenia and talks a lot; Paternal grandmother has auditory problem	1

109	after 2 miscarriage during pregnancy she took iron health tablet for anemia	1	Mother is suffering from Bipolar disorder and has high suicidal tendency; Paternal uncle had Schizophrenia	1
110	None	0	Paternal uncle had delayed speech	1
111	None	0	Mother had history of depression during pregnancy	1
112	excessive vomitting & sickness in mother during pregnancy, cough & cold, fainting due to high fever	1	elder sister autistic	1
113	cough & cold during pregnancy	1	father had delayed speech & language development history,paternal aunt also talk less	1
114	she had contraceptive medication four months before pregnancy,convulsion during fever two or three times	1	mother had depression throughout pregnancy	1
115	None	0	maternal uncle had mental retardation and passed away at 54 years,	1

116	jaundice	1	father has delayed speech,socially withdrawn at early stages,father cousin son had also delayed speech,premature 34 weeks & premature rupture of membrane during this childbirth	1
117	at 7th month, had asprin because of high BP	1	mother allergic to brinjal,nephew of mother has dyslexia,mother had primary post partum haemorrhage in breast,mental illness in mother's dside	1
118	None	0	None	0
119	None	0	None	0
120	None	0	None	0
121	None	0	None	0
122	None	0	delayed speech in mother's paternal aunts sister son	1
123	after two month of conceiving she got movement restriction and had chances of miscarriage for which she was taken gestofid.	1	delayed speech of father & paternal uncle,mother's maternal cousin sister had mental illness	1

124	immediately after delivery she got affected with gland TB	1	schizophrenia in maternal grandmother,mental retardation in mother's family,mother on thyroid problems throughout pregnancy	1
125	None	0	None	0
126	None	0	None	0
127	None	0	None	0
128	None	0	paternal uncle had difficulty in speech,father has high bp	1
129	hypertension	1	mother had mental retardation in childhood	1
130	None	0	mother had delayed walking history	1
131	None	0	None	0
132	None	0	maternal uncle is hyperactive & has less concentration	1
133	None	0	delayed speech in father, paternal cousin	1
134	None	0	None	0
135	None	0	None	0
136	None	0	None	0
137	None	0	grand uncle has intellectual disability	1
138	None	0	None	0

139	taken novalgene before pregnancy, thyroid problem during giving birth	1	epilepsy in father,ocd in paternal grandmother	1
140	jaundice at 7th months	1	mental retardation in mothers paternal uncle	1
141	None	0	mental illness in maternal uncle	1
142	None	0	mental illness in maternal grandmother	1
143	pregnancy was delayed, she has fibroid	1	None	0
144	None	0	mental retardation in father's family,delayed speech in father's family	1
145	mother had thyroid problem, spotting (initially, it was 2 months)	1	None	0
146	high bp, arthritis and itching problems, obesity, severe rheuatoid artheritis	1	None	0
147	None	0	None	0
148	nausea during 1st trimester, fever during last trimester	1	None	0
149	movement of the fetus was restricted	1	delayed speech in father	1

150	bedridden during the time of pregnancy as she had prior miscarriages	1	delayed speech and delayed developmental procedures in maternal aunt	1
151	suffer lot of pain during child birth	1	None	0
152	first pregnancy was destroyed and 2nd was IVF	1	paternal uncle had autism	1
153	None	0	None	0
154	under medication due to some pregnancy problem	1	delayed speech in father	1
155	None	0	delayed speech in maternal uncle	1
156	None	0	fathers elder sister was mentally retarded	1
157	mid trimester she had high bp	1	delayed speech in paternal cousin	1
158	mother had malaria before conceving and could not complete the course of medication	1	father had delayed speech	1
159	None	0	fathers paternal uncle had mental illness at the age of 40, maternal cousin has autism	1

160	severe constipation, pressuised bowel evacuation during pregnancy	1	maternal cousin had autism	1
161	None	0	None	0
162	None	0	None	0
163	mother had fibroid during pregnancy	1	delayed speech in paternal uncle and grand mother	1
164	hypertension	1	None	0
165	None	0	None	0
166	Bleeding at 3 months	1	maternal cousin brother mentally retarded	1
167	none	0	None	0
168	None	0	delayed speech in mother's family	1
169	Exchange blood transfusion to the mother.	1	Delayed speech in parental cousin	1
170	NONE	0	None	0
